# Serogroup-Specific Characteristics of Localized Meningococcal Meningitis Epidemics in Niger 2002–2012 and 2015: Analysis of Health Center Level Surveillance Data

**DOI:** 10.1371/journal.pone.0163110

**Published:** 2016-09-22

**Authors:** Halima Boubacar Maïnassara, Juliette Paireau, Issa Idi, Jean-François Jusot, Jean-Paul Moulia Pelat, Odile Ouwe Missi Oukem-Boyer, Arnaud Fontanet, Judith E. Mueller

**Affiliations:** 1 Centre de Recherche Médicale et Sanitaire, Niamey, Niger; 2 Institut Pasteur, Emerging Diseases Epidemiology Unit, Paris, France; 3 Université Pierre et Marie Curie, Cellule Pasteur UPMC, Paris, France; 4 Department of Ecology and Evolutionary Biology, Princeton University, Princeton, NJ, United States of America; 5 Conservatoire National des Arts et Métiers, Chaire Santé et Développement, Paris, France; 6 EHESP French School of Public Health, Sorbonne Paris Cité, Rennes, France; Universidad Nacional de la Plata, ARGENTINA

## Abstract

To compare dynamics of localized meningitis epidemics (LE) by meningococcal (Nm) serogroup, we analyzed a surveillance database of suspected and laboratory-confirmed Nm cases from 373 health areas (HA) of three regions in Niger during 2002–2012 and one region concerned by NmC epidemics during 2015. We defined LE as HA weekly incidence rates of ≥20 suspected cases per 100,000 during ≥2 weeks and assigned the predominant serogroup based on polymerase chain reaction testing of cerebrospinal fluid. Among the 175 LE, median peak weekly incidence rate in LE due to NmA, W, X and C were 54, 39, 109 and 46 per 100,000, respectively. These differences impacted ability of the epidemic to be detected at the district level. While this analysis is limited by the small number of LE due to NmX (N = 4) and NmW (N = 5), further research should explore whether strategies for prevention and response to meningitis epidemics need to be adapted according to predominant meningococcal serogroups.

## Introduction

In the African meningitis belt, major epidemics of bacterial meningitis were historically due to the meningococcus of serogroup A. Since the introduction of the conjugate vaccine against this predominant epidemic agent, (PsA-TT, MenAfrivac®), the overall incidence of suspected cases of acute bacterial meningitis has declined in all vaccinated countries and meningococcal (Nm) serogroup A cases have been identified only exceptionally [1]. However, other meningococcal serogroups such as W, X and C can still cause outbreaks [2, 3–5]. NmW, whose first documented outbreak occurred in Burkina Faso in 2002 [6], has been the most frequently identified serogroup since PsA-TT introduction [1, 2]. With no available vaccine against that serogroup, NmX is also a threat, as shown by the past outbreaks reported in Ghana in 2000 [4], in Niger in 1990 [7, 8] and 2006 [9], in Togo in 2007 and in Burkina Faso in 2010 [5]. NmC, which occurred infrequently in the meningitis belt, re-emerged during an outbreak in Nigeria in 2013–2014 [10] and in Niger in 2015 (unpublished observations), whereas the last outbreak in the region dated back to 1979 [11].

Despite this risk related to other serogroups and their relatively increased importance with the reduction of NmA meningitis, no analysis of epidemic dynamics of these serogroups in comparison to NmA has been reported so far, in particular spanning a longer period and at fine spatial level. It therefore appears important to explore whether the other serogroups show similar epidemic dynamics to NmA. This will help the policy makers to adjust the response strategies accordingly, such as the definition of alert and epidemic thresholds and the optimal spatial level of surveillance and reactive vaccination.

Following a hypothetical model proposed by Mueller & Gessner [12], several studies now have confirmed that within epidemic districts, the epidemic hotspots are usually highly localized around a few health centers, while most other health centers remain in non-epidemic situation [13, 14]. In consequence, to understand the epidemic dynamics of different serogroups, analysis of surveillance data at fine spatial resolution is required [14]. While most meningitis belt countries lack such fine data, we were able to use, for the present analysis, suspected case report data collected in Niger at the health area (HA) level, which had the additional advantage of including laboratory confirmation of cases. In this paper, we aim to compare dynamics of localized epidemics by serogroup in Niger, at fine spatial resolution during 2002–2012 and 2014–2015.

## Methods

### Databases

Data on suspected bacterial meningitis cases were collected during 2002–2012 and 2014–2015 in Nigerien health centers, for routine country-wide epidemiological surveillance. To analyze epidemic dynamics at the HA level, we aggregated the original health center case counts at the HA level and selected three regions (Tahoua, Tillabery and Dosso) for analysis as described in details previously [15]. A map of the study area, with limits of regions, districts and health areas is available in S1 Fig. No data was collected in 2012–2013 and 2013–2014 since no epidemic occurred in Niger [16]. The 2014–2015 data were collected in Dosso region only, due to logistic and financial constraints. Dosso region was one of the principal setting of the NmC epidemics in Niger during 2014–2015.

Data on confirmed meningococcal cases were collected and merged with the suspected cases database as described elsewhere [15]. Based on these data, we could identify the causal serogroup of each localized meningitis epidemic.

The Niger national ethics committee approved this research (N° 014/2012/CCNE).

### Statistical analysis

We calculated weekly incidence rate and annual incidence for each HA, as defined previously [15]. To group cases belonging to the same meningitis season (running from November 1^st^ through May), we defined an epidemiological year *n* from 1^st^ July of calendar year *n-1* to 30^th^ June of calendar year *n*. All analysis were performed in R Studio software version 2.15.3 [17]. Maps were created with QGIS software version 1.8.0 [18].

Localized epidemics (LE) of suspected cases were identified using a method previously described by Tall [13]. We chose as the primary reference standard an annual incidence above the 95^th^ percentile of annual incidences in all HA in the database (130 per 100,000) and as the secondary reference an annual incidence above the 97.5^th^ percentile (210 per 100,000). We tested fourteen thresholds of weekly incidence rates at the HA level to define a LE, ranging from 5 to 200 cases per 100,000 during at least two consecutive weeks, named LE5, LE10 etc. We added the requirement of two consecutive weeks to limit bias due to operational issues such as non-continuous reporting. The optimal thresholds were chosen on a receiver-operator curve (ROC) with regard to their sensitivity and specificity in detecting HA with eventual annual incidence ≥130 per 100,000 and ≥210 per 100,000. We compared LE of serogroup A versus LE of serogroups W, LE of serogroup A versus LE of serogroup X, LE of serogroup A versus LE of serogroup C and then compared serogroup W versus serogroup X. To test for robustness of analyses in small health areas where one or two cases could represent a localized epidemic, analyses of LE characteristics were also performed for LE with population size <30,000 with the requirement of at least 3 weekly cases; and specifically for health areas with ≥30,000 in order [19]. The test of Mann-Whitney was used to determine whether medians of LE characteristics were different between serogroups. In case of equally placed values, we used Kruskall Wallis test. The correction of continuity was used to reduce biases due to small samples. We compared the durations of LE (defined as the number of weeks between the first and the last weeks of the threshold crossing) by using the Chi-2 test or the Fisher’s exact test. To assess the degree of clustering or dispersion of the LE across geographic space, spatial autocorrelation was measured by the joint count statistics for each year. Differences with a *P*-value ≤ 0.05 were considered significant.

## Results

**Fig 1:**
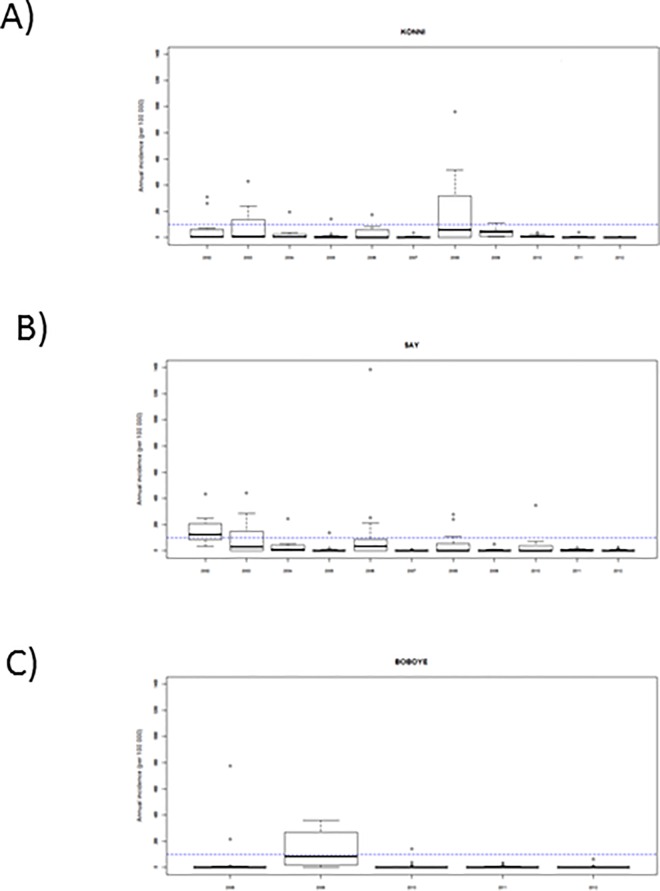
Annual meningitis incidences at health area level in three selected districts, Niger, 2002–2012. One district was selected for illustration in each of the three regions: A) Konni district in Tahoua region, B) Say district in Tillabery region, and C) Boboye district in Dosso region. Boxplots show median, 25^th^ and 75^th^ percentile and range of annual incidences in the health areas of the district. Dotted lines indicate annual incidence of 100 per 100,000, a threshold previously used as a retrospective definition of epidemics at the district level.^13^ The epidemiological year *n*, from 1^st^ July of calendar year *n-1* to 30^th^ June of calendar year *n*, is used here.

From 2002 to 2012, we included 154,392 weekly HA reports, corresponding to 3,357 health area years, with 14,921 suspected meningitis cases. During this period, 7,377 cerebrospinal fluids of Tahoua, Dosso, Tillabery regions were sent to CERMES and analyzed by polymerase chain reaction. Among these, 2,224 cases were due to NmA, 224 to NmX, 495 to NmW, 83 to Hi, 446 to *Streptococcus pneumoniae* (Sp) and 3,905 were negative. We found substantial heterogeneity in annual incidences between HA of the same district (Fig 1A, 1B and 1C). At the HA level, the highest annual incidences observed in each region were 1384 cases per 100,000 inhabitants in the district of Say, 960 per 100,000 in the district of Konni and 780 per 100,000 in the district of Boboye. From 1^st^ July 2014 to 30 June 2015, we included 4,763 weekly HA reports collected in 91 HA of the five districts of Dosso region with 1,282 suspected cases. During this period, 819 cerebrospinal fluids sent from Dosso region were analyzed by polymerase chain reaction. Among these, there were 372 NmC cases, 69 NmW, 24 Sp and 354 negative results.

**Fig 2:**
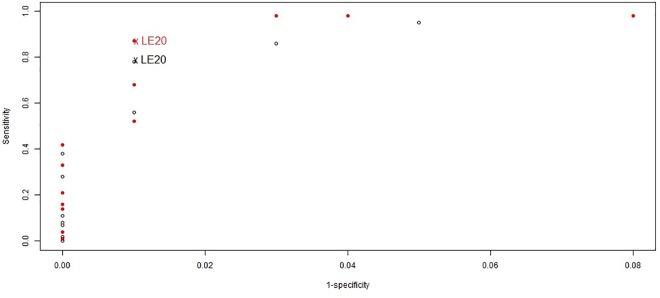
Performance of localized epidemic definitions in detecting elevated annual meningitis incidences at the health area level; in Tahoua, Tillabery and Dosso regions, Niger, 2002–2012 (3,357 health area years). Empty marks represent the performance in detecting annual incidences ≥130 cases per 100,000 which corresponds to the 95^th^ percentile of all annual incidences in the database. Full and red marks represent the performance in detecting annual incidences ≥210 cases per 100,000 inhabitants which corresponds to the 97.5^th^ percentile of all annual incidences in the database. Definitions for localized epidemics are based on different thresholds of weekly incidence rates per 100,000 inhabitants (5, 10, 15, 20, 30, 40, 50, 60, 70, 80, 90, 100, 150, 200 suspected cases) with incidences maintained during at least two weeks. LE20 indicates the threshold retained for further analyses.

When the definition of LE varied from 5 to 200 weekly cases per 100,000 (during ≥2 weeks), sensitivity to detect HA with annual incidence ≥130 per 100,000 (95^th^ percentile of annual incidences) varied from 95% to 0% and specificity from 95% to 100%; sensitivity to detect HA with annual incidence ≥210 per 100,000 (97.5^th^ percentile of annual incidences) varied from 98% to 0.01% and specificity from 92% to 100% (Fig 2). For further analyses, we chose the threshold of 20 cases per 100,000 per week crossed during ≥2 consecutive weeks, which had a sensitivity of 78% and a specificity of 99% in detecting an annual incidences ≥130 per 100,000 with a positive predictive value of 81% and a negative predictive value of 99%.

We thus identified 162 LE during 2002 to 2012, with the annual number of LE ranging from 0 during 2007 and 2012, to 88 during 2009 (Table 1). LE were due to NmA (N = 98, 60%), NmW (N = 4) and NmX (N = 4) based on the predominant serogroup found in the HA, while 56 (35%) had no principal aetiology defined (mainly due to lack of laboratory investigation). From July 1 2014 to June 30 2015, we identified 13 LE among the 91 HA of Dosso region, which were due to NmC (N = 9) and NmW (N = 1), while no laboratory investigation was available for 3 LE (23%).

**Table 1:** Characteristics of meningitis localized epidemics at the health area level in Niger, by epidemic agent. Tahoua, Tillabery and Dosso regions, 2002–2012 and Dosso region, July 2014-June 2015.

	Identification of LE			Epidemic force of LE		Temporal description of LE	
	Number of HA with LE	Number of suspected meningitis cases during the week when the LE definition was met	Population size in the HA with LE (/10^3^)	Annual incidence in the HA with LE	Peak weekly incidence in the HA with LE	LE duration in weeks	Calendar week when the LE definition was met
**Tahoua, Tillabery and Dosso regions, 2002–2012**							
Serogroup A	98	7 (1–44)	18.4 (4.6–102.9)	220 (73–959)	54 (21–287)	4 (2–13)	12 (41ª–18)
Serogroup W	4	7 (2–13)	20.7 (5.9–61.2) †	127 (108–464) †	39 (28–172) †	2 (2–3) §	11 (7–14) †
Serogroup X ‡	4	5 (2–6)	11.5 (5.9–21.4) †	252 (115–1384) †	109 (21–614) †	3 (2–8) §	10 (6–15) †
Other LE *	56	3 (1–18)	9.6 (3.8–43.6)	191 (44–777)	59 (21–475)	3 (2–11)	13 (3–19)
**Dosso region, 2014–2015**							
Serogroup C	9	8 (5–22)	31.3 (13.9–52.9) †	172 (96–624) †	46 (33–168) †	3 (2–8) §	17 (12–18) †
Serogroup W	1	15	39.9	115	42	2	18
Other LE **	3	12 (4–16)	18.5 (14.9–32.9)	130 (59–475)	45 (21–220)	3 (2–4)	19 (18–21)

Localized epidemics were defined as weekly incidence at the HA level ≥20 per 100,000 during ≥2 consecutive weeks. Figures are median (range). Annual and weekly incidences are rates per 100,000. LE, localized epidemic; HA, health area corresponding to the population served by one health center. The calendar week is defined from week 1 to week 52 of the calendar year *n*.

* localized epidemics without laboratory investigation (N = 48), with equal presence of several meningococcal serogroups (N = 4) or exclusively etiology-negative samples of cerebrospinal fluid (N = 4)

** localized epidemics without laboratory investigation, occurred all in the same district

† *P*<0.001 for difference to serogroup A

‡ *P*>0.05 for difference between serogroups W and X for all indicators

§ *P*>0.05 for difference to serogroup A

ª Week of the calendar year *n*-1

**Fig 3:**
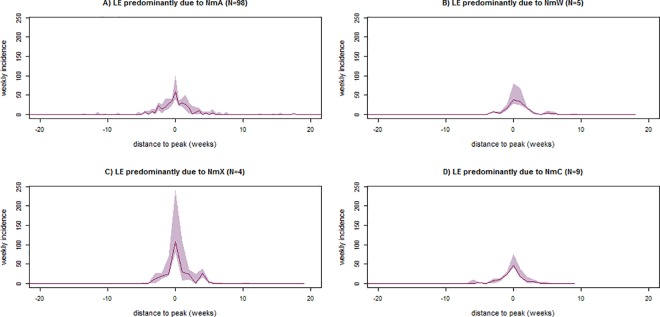
Epidemic curves (weekly incidence rates per 100,000) of suspected meningitis cases in health areas with localized epidemics (LE), by epidemic serogroups, in Tahoua, Tillabery and Dosso regions, Niger, 2002–2012 and 2014–2015. The localized epidemics were defined with the threshold of 20 cases per 100,000 inhabitants during at least two consecutive weeks (LE20). The solid line represents the median, and the shaded area represents the 25^th^ and 75^th^ percentiles of the weekly incidences. Epidemic agents were defined according to the majority of pathogens identified by systematic polymerase chain reaction testing on cerebrospinal fluid.

At the HA level, LE due to NmX presented substantially higher median peak weekly incidence rate than LE due to NmA (109 vs. 54 per 100,000; p<0.001) (Table 1), while the difference in median annual incidence were less pronounced (252 vs. 220 per 100,000; p<0.001). Compared to other serogroups, LE due to NmW presented lower median peak weekly incidence rate (vs. NmA, 39 vs. 54 per 100,000; p<0.001) and median annual incidence (vs. NmA, 127 vs. 220 per 100,000; p<0.001). The median peak weekly incidence rate and annual incidence of LE due to NmC during 2014–2015 (46 and 172 per 100,000, respectively) were higher than those due to NmW during the same or the 2002–2012 period, and lower than NmA or NmX during the 2002–2012 period (Table 1). The epidemic curves of LE due to different serogroups had similar shapes (Fig 3), while the peak of LE due to NmX appeared to be higher and be reached more quickly.

Differences between serogroups were similar at the district level: NmX appeared to have higher median peak weekly incidence rate than other serogroups, while median annual incidence was similar to NmA and NmC (Table 2). NmC showed median peak weekly incidence rate and annual incidence similar to NmA. NmW showed lower median peak weekly incidence rate and annual incidence than other serogroups. In most LE, the LE definition was met when district level weekly incidence rate were below the WHO-recommended threshold for epidemic response of 10 per 100,000 [19] (median district weekly incidence rate 8, range 0.2–30). Visibility of the epidemics (i.e ability to be detected at the district level) was better for NmA (median district weekly incidence rate 9 per 100,000, range 1–30) than NmW (5, 3–7) or NmX (4,1–6). The duration of LE tended to be longer for NmA (median 4 weeks) compared to the other serogroups (median 2 or 3 weeks) (all p>0.05) (Table 1). Detection of LE occurred substantially later during 2014–15 compared to 2002–2012, with little difference between serogroups (Table 1).

**Table 2:** Visibility of meningitis localized epidemics (LE) in Niger at the district level, by epidemic agent in health areas (HA). Tahoua, Tillabery and Dosso regions, 2002–2012 and Dosso region, July 2014-June 2015.

	Number of HA with LE	Total population concerned by LE in the district (/10^3^)	Annual incidence in the districts with LE	Peak weekly incidence in the districts with LE	Weekly incidence in the district when the LE definition was met
**Tahoua, Tillabery and Dosso regions, 2002–2012**					
Serogroup A	98	47.6 (4.6–276.7)	84 (21–179)	16 (4–34)	9 (1–30)
Serogroup W	4	20.7 (5.8–61.2)	42 (40–44)	7 (5–9)	5 (3–7)
Serogroup X	4	25.2 (6.0–44.4)	89 (24–155)	50 (6–50)	4 (1–6)
Other LE *	56	14.1 (4.2–87.5)	56 (7–166)	12 (1–27)	7 (0.2–26)
All LE combined	162	32.6 (4.2–276.7)	54 (7–179)	15 (1–50)	8 (0.2–30)
**Dosso region, 2014–2015**					
Serogroup C	9	97.8 (29.7–146.7)	73 (43–86)	17 (12–26)	7 (3–10)
Serogroup W	1	39.9	23	8	8
Other LE **	3	66.4	43	17	6 (4–17)
All LE combined	13	96.9 (39.9–146.7)	58 (23–86)	17 (8–26)	8 (3–17)

Localized epidemics were defined as weekly incidence at the HA level ≥20 per 100,000, maintained during ≥2 weeks. Figures are median (range). Annual and weekly incidences are rates per 100,000. LE, localized epidemic; HA, health area corresponding to the population served by one health center

* localized epidemics without laboratory investigation (N = 48), with equal presence of several meningococcal serogroups (N = 4) or exclusively etiology-negative samples of cerebrospinal fluid (N = 4)

** localized epidemics without laboratory investigation, occurred all in the same district

Localized epidemics occurred in relatively small populations: The median size of HA populations affected by a LE was 14,420 (range 3,799–102,900), corresponding in median to 5% (range 1–24%) of the district population. The median population of health areas where an LE due to NmA was identified was larger than those due to NmX (18,400 vs. 11,500; p<0.001), but smaller than those due to NmW (18,400 vs. 20,700; p<0.001) or NmC (18,400 vs. 31,300; p<0.001). Most LE were identified based on 5 or more suspected cases occurring during the second of the two consecutive weeks with weekly incidence rates ≥20 per 100,000, but 18% (31/175) were identified based on only 1 or 2 cases.

When restricting the analyses to LE defined in small health areas with <30,000 inhabitants and based on ≥3 cases per week, 105 and 6 LE were identified during 2002–2012 and 2014–15, without only one LE due to NmW (S1 Table). Median peak weekly incidence rate were highest for NmX, while differences in median annual incidence were small. Analyses restricted to LE in large health areas with ≥30,000 inhabitants identified 26 and 7 LE, respectively, during the two periods, without any LE due to NmX (S2 Table). Median peak weekly incidence rate were lowest for NmW, while median annual incidence was highest for NmA.

**Fig 4:**
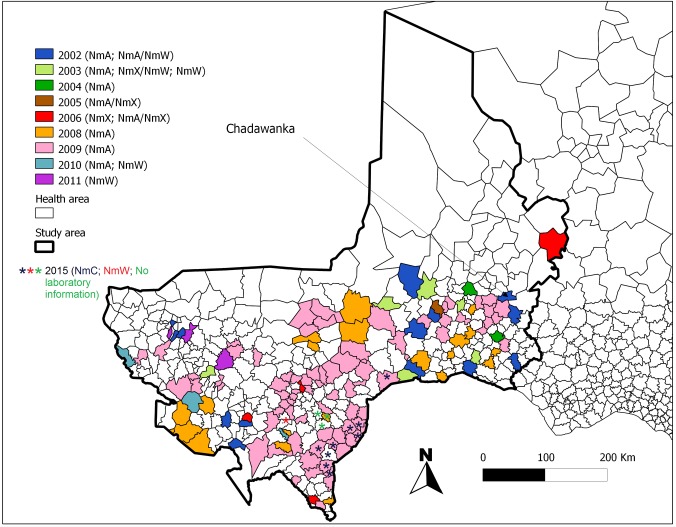
Spatial distribution and epidemic agent of the 175 meningitis localized epidemics in health areas of Tahoua, Tillabery and Dosso regions, Niger, 2002–2012 and 2014–2015. A localized epidemic was defined at the health area level as 20 suspected cases per 100,000 during at least two consecutive weeks (LE20). The health area of Chadawanka was the only HA which experienced four times a localized epidemic in consecutive years 2002–2003 and 2005–2006. Epidemic agents were defined according to the majority of pathogens identified by systematic polymerase chain reaction testing on cerebrospinal fluid. The epidemiological year *n*, from 1^st^ July of calendar year *n-1* to 30^th^ June of calendar year *n*, is used here.

Localized epidemics across the whole study period were located towards the South of the study area and nearby the frontier to Nigeria, where the population density is greatest (Fig 4 and S1 Fig). Among the 162 HA of 2002–2012, 10 (6%) experienced two or three times an LE. Chadawanka in Abalak district experienced LEs four times: during 2002 (serogroup unknown), 2003 (NmA), 2005 (NmA and X) and 2006 (NmA). The LE did not follow any systematic spatiotemporal order of occurrence. During 2008 and 2009 (two years of particularly intensive incidence in Tahoua and Dosso regions), localized epidemics due to NmA showed positive spatial autocorrelation (i.e. these LE were more spatially clustered than expected by chance), while during other years, LE of any serogroup followed a random spatial pattern (no significant spatial autocorrelation).

## Discussion

In this description of serogroup-specific dynamics of meningitis epidemics at fine spatial scale in Niger, we found differences in peak epidemic force and cumulative annual incidence between serogroups. In particular, we found that epidemics due to NmX tended to have higher peak force at health center level than those due to serogroup A, while the peak force of NmW appeared to be lower. The re-emerging serogroup C appeared to have epidemic dynamic similar to NmA. Annual incidences showed less pronounced differences, possibly due to the trend for longer duration of NmA epidemics.

Mueller & Gessner [12] hypothesized that meningitis LE can occur with any serogroup as soon as epidemic co-factors increasing carriage prevalence (which largely remain unknown) are present, while the epidemic serogroup responsible would be the one predominantly carried at that time. Carriage studies during epidemics both due to NmA or NmW have found exceptionally high carriage prevalences [20, 21], compatible with this hypothesis. If the model holds for NmX as well, differences between serogroups in terms of epidemic force, as found in the present analysis, may represent differential transmission dynamics or differential interaction with epidemic co-factors. These differences between serogroups could be due to differences between meningococcal clonal complexes involved in meningitis over the observed period. Sequence types (ST) within the ST-5 clonal complex, associated with NmA, and the ST-11 clonal complex, associated with NmW, are believed to be more virulent (higher odds of invasive disease given carriage) than NmX, at least that of ST-751 [4]. However, as pointed out by Boisier et al. [3], the ST-181 clonal complex, responsible of all epidemic NmX events in West Africa since 2005–06, may be more virulent than other clonal complexes, despite its genetic proximity with ST-751. As in all ecological analyses, we cannot exclude that several unobserved coinciding factors which caused the observed differences in serogroup dynamics, such as population behaviour or environmental conditions. However, all localized epidemics identified for NmX occurred during 2005–2006, when NmX and A co-existed in the epidemic regions of Niger [3], suggesting that such coinciding factors would not explain serogroup differences. By contrast, during 2015, when the LE due to NmC were observed with some cases of NmW, NmA was absent following PsA-TT introduction. As interactions between meningococcal serogroups in terms of transmission and circulation in populations are largely unexplored, caution is required when comparing epidemic dynamics of serogroups in the presence and absence of serogroup A.

The fact that most LE were identified before district level incidences crossed the threshold for epidemic response, confirms the utility of analysing data at the subdistrict level, as recommended by WHO (30,000 inhabitants) [19]. Using the same data from Niger, we recently showed that in a simulated scenario of NmA elimination, surveillance and vaccination response would substantially gain in effectiveness and efficiency if conducted at the subdistrict level [15]. Further surveillance and analysis is needed to refine such recommendations for specific predominant serogroups, as currently, only vaccines with limited serogroup coverage and limited stocks are available and affordable for the meningitis belt while impact on epidemics depends on rapid and serogroup-specific response.

Surveillance in small populations increases the risk of false positive signals due to wide confidence intervals around incidence estimates, which may have biased the differences between serogroups reported here. However, additional analyses in health areas of <30,000 inhabitants and requesting at least three weekly cases for identification of localized epidemics showed a similar pattern of differences between serogroup-specific dynamics. Our results support the seemingly randomly-defined epidemic threshold of 5 cases / 100,000 inhabitants in populations <30,000 inhabitants. Our LE definition required two consecutive weeks of incidences above the threshold, which probably avoided false positive signals and rendered the definition sufficiently specific.

Our analysis has several limitations. We selected only three regions in Niger, in which data exhaustiveness was satisfactory. Our analyses may therefore have limited external validity. Furthermore, some discrepancy persisted between the two databases and reporting from epidemic district weeks may have been more accurate; however, this difference was not substantial and suggested both over- and underestimation in a non-differential manner. Although the polymerase chain reaction-based surveillance system in Niger stipulates that all suspected cases should undergo lumbar puncture and all cerebrospinal fluid samples are sent for analysis, 51 LE (more than one third) in our analysis remained without etiological information due to missing laboratory analysis. The highest proportions of LE without laboratory information were found during epidemiological years 2002 and 2003 when polymerase chain reaction-based surveillance was introduced in Niger. Smaller or atypical epidemic events may be associated with absence or partial laboratory investigation, and more remote health centers may have encountered greater difficulties with regard to logistics or human and financial resources. Other localized epidemics could have been due to pneumococcus. Polymerase chain reaction testing in Niger has been reported to have limited sensitivity for detection of NmA and NmW during some periods [22], with substantial proportions of false aetiology-negative cases. A part of the LE classed as “negative” may therefore in fact be due to NmA or NmW, which may have led to over- or underestimation of serogroup-specific incidences.

Overall, this analysis is in particular limited by the small number of localized epidemics due to NmX and NmW that could be observed, and the impossibility to control for other coinciding factors influencing epidemic dynamics. However, these data represent a rare opportunity to conduct such a serogroup-specific analysis, by combining suspected and confirmed meningitis surveillance data at fine spatial resolution over more than a decade and several regions. This analysis provides additional information to reports on epidemics due to NmW, NmX and NmC [2–5, 10], and suggests that strategies for prevention and epidemic response may need to be adapted according to predominant serogroups. It underpins that the different meningococcal serogroups must all be considered as major threat for meningitis epidemics after elimination of serogroup A.

## Supporting Information

S1 Table Characteristics of meningitis localized epidemics at the health area level in Niger, by epidemic agent, including only health areas with <30,000 inhabitants. Tahoua, Tillabery and Dosso regions, 2002–2012 and Dosso region, July 2014-June 2015.
(DOCX)

S2 Table Characteristics of meningitis localized epidemics at the health area level in Niger, by epidemic agent, including only health areas with ≥30,000 inhabitants. Tahoua, Tillabery and Dosso regions, 2002–2012 and Dosso region, July 2014-June 2015.
(DOCX)

S1 Fig Map of Niger, showing borders of health areas, districts and regions. **The yellow part corresponds to the study area and encompasses the three regions of Tahoua, Tillabery and Dosso**. Health areas were defined based on the geographical locations of health centers and their surrounding villages, and therefore do not always strictly match the borders of the administrative regions.
(TIFF)

S1 Supporting Information Weekly suspected meningitis cases in 2014–2015. (XLS)

S2 Supporting Information Weekly confirmed meningitis cases in 2002–2012. (XLS)

S3 Supporting Information Weekly confirmed meningitis cases in 2014–2015. (XLS)

S4 Supporting Information Weekly suspected meningitis cases in 2002–2012. (XLS)
