# Supplementary material for: Serogroup-Specific Characteristics of Localized Meningococcal Meningitis Epidemics in Niger 2002–2012 and 2015: Analysis of Health Center Level Surveillance Data
Source: PLoS One. 2016 Sep 22;11(9):e0163110. doi: 10.1371/journal.pone.0163110 (PMC5033479; doi:10.1371/journal.pone.0163110)
Supplement: S1 Table — Tahoua, Tillabery and Dosso regions, 2002–2012 and Dosso region, July 2014-June 2015. (DOCX) [file pone.0163110.s001.docx]

S1 Table. **Characteristics of meningitis localized epidemics at the health area level in Niger, by epidemic agent, including only health areas with <30,000 inhabitants.** Tahoua, Tillabery and Dosso regions, 2002-2012 and Dosso region, July 2014-June 2015.

|  | **Identification of LE** | | | | | | **Epidemic force of LE** | | | | | | **Temporal description of LE** | | | |
| --- | --- | --- | --- | --- | --- | --- | --- | --- | --- | --- | --- | --- | --- | --- | --- | --- |
|  | Number of HA with LE | Number of suspected meningitis cases during the week when the LE definition was met | | | Population size in the HA with LE (/10^3^) | | Annual incidence  in the HA with LE | | Peak weekly incidence in the HA with LE | | LE duration in weeks | | | | Calendar week when the LE definition was met | |
| **Tahoua, Tillabery and Dosso regions, 2002-2012** | | | |  | |  | | | |  | |  | |  | |  |
| Serogroup A | 67 | | 6 (3 – 30) | 16.4 (4.9 – 29.5) | | 215 (79 – 959) | | 61 (21 – 287) | | | | 4 (2 – 11) | | 12 (46ª –18) | |  |
| Serogroup W | 1 | | 5 | 10.2 † | | 108 † | | 49 † | | | | 2 § | | 9 † | |  |
| Serogroup X | 3 | | 5 (5 – 6) | 13.4 (5.9 – 21.4) † | | 252 (250 – 1384) † | | 117 (100 – 614) † | | | | 4 (3 – 8) § | | 11 (10 – 15) † | |  |
| Other LE * | 34 | | 6 (3 – 16) | 13.2 (5.5 – 29) | | 193 (44 – 501) | | 60 (22 – 201) | | | | 2 (2 – 11) | | 13 (5 – 17) | |  |
| **Dosso region, 2014-2015** | | | |  | |  | |  | | | |  | |  | |  |
| Serogroup C | 4 | | 7 (5 – 22) | 17.4 (13.9 – 29.7) † | | 225 (135 – 624) † | | 104 (41 – 169) † | | | | 3 (2 – 8) § | | 17 (12 – 18) † | |  |
| Other LE ** | 2 | | 10 (4 – 16) | 16.7 (14.9 – 18.5) | | 267 (59– 475) | | 121 (21 – 221) | | | | 3 (2 – 4) | | 19 (18 – 21) | |  |

Localized epidemics were defined as weekly incidence at the HA level ≥20 per 100,000 during ≥2 consecutive weeks, **with ≥3 cases per week**.

Figures are median (range). Annual and weekly incidences are rates per 100,000

LE, localized epidemic; HA, health area corresponding to the population served by one health center

* localized epidemics without laboratory investigation (N=48), with equal presence of several meningococcal serogroups (N=4) or exclusively etiology-negative samples of cerebrospinal fluid (N=4)

** localized epidemics without laboratory investigation , occurred all in the same district

† *P*<0.001 for difference to serogroup A; § *P*>0.05 for difference to serogroup A

ª Week of the calendar year *n*-1

The calendar week is defined from week 1 to week 52 of the calendar year *n*.
