# Supplementary material for: Serogroup-Specific Characteristics of Localized Meningococcal Meningitis Epidemics in Niger 2002–2012 and 2015: Analysis of Health Center Level Surveillance Data
Source: PLoS One. 2016 Sep 22;11(9):e0163110. doi: 10.1371/journal.pone.0163110 (PMC5033479; doi:10.1371/journal.pone.0163110)
Supplement: S2 Table — Tahoua, Tillabery and Dosso regions, 2002–2012 and Dosso region, July 2014-June 2015. (DOCX) [file pone.0163110.s002.docx]

S2 Table. **Characteristics of meningitis localized epidemics at the health area level in Niger, by epidemic agent, including only health areas with ≥30,000 inhabitants.** Tahoua, Tillabery and Dosso regions, 2002-2012 and Dosso region, July 2014-June 2015.

|  | |  | | | |  |  |  |  |  |  |
| --- | --- | --- | --- | --- | --- | --- | --- | --- | --- | --- | --- |
|  | | Number of HA with LE | Number of suspected meningitis cases during the week when the LE definition was met | | | | Population size in the HA with LE (/10^3^) | Annual incidence  in the HA with LE | Peak weekly incidence in the HA with LE | LE duration in weeks | Calendar week when the LE definition was met |
| **Tahoua, Tillabery and Dosso regions, 2002-2012** | | | | | | |  |  |  |  |  |
| Serogroup A | 23 | | | | 14 (8 – 44) | | 39.9 (30.3 – 102.9) | 202 (75 – 428) | 40 (21 – 136) | 3 (2 – 8) | 12 (4 –18) |
| Serogroup W | 2 | | | | 11 (9 – 13) | | 46.3 (31.3 – 61.2) § | 127 (118 – 136) § | 28 (28 – 29) § | 2 (2 – 3) § | 14 (14 – 14) § |
| Other LE * | 1 | | | | 18 | | 43.6 | 144 | 41 | 3 | 12 |
| **Dosso region, 2014-2015** | | | | | | |  |  |  |  |  |
| Serogroup C | 5 | | | 9 (7 – 12) | | | 34.9 (31.3 – 52.9) § | 123 (96 – 219) § | 41 (33 – 77) § | 2 (2 – 3) § | 17 (16 – 18) † |
| Serogroup W | 1 | | | 15 | | | 39.9 | 115 | 42 | 2 | 18 |
| Other LE * | 1 | | | 12 | | | 32.9 | 130 | 45 | 3 | 19 |

Localized epidemics were defined as weekly incidence at the HA level ≥20 per 100,000 during ≥2 consecutive weeks.

Figures are median (range). Annual and weekly incidences are rates per 100,000

LE, localized epidemic; HA, health area corresponding to the population served by one health center

* localized epidemics without laboratory investigation

§ *P*>0.05 for difference to serogroups A; † *P*<0.01 for difference to serogroup A ;

The calendar week is defined from week 1 to week 52 of the calendar year *n*.
