# Supplementary figures and images for: Serogroup-Specific Characteristics of Localized Meningococcal Meningitis Epidemics in Niger 2002–2012 and 2015: Analysis of Health Center Level Surveillance Data
Source: PLoS One. 2016 Sep 22;11(9):e0163110. doi: 10.1371/journal.pone.0163110 (PMC5033479; doi:10.1371/journal.pone.0163110)

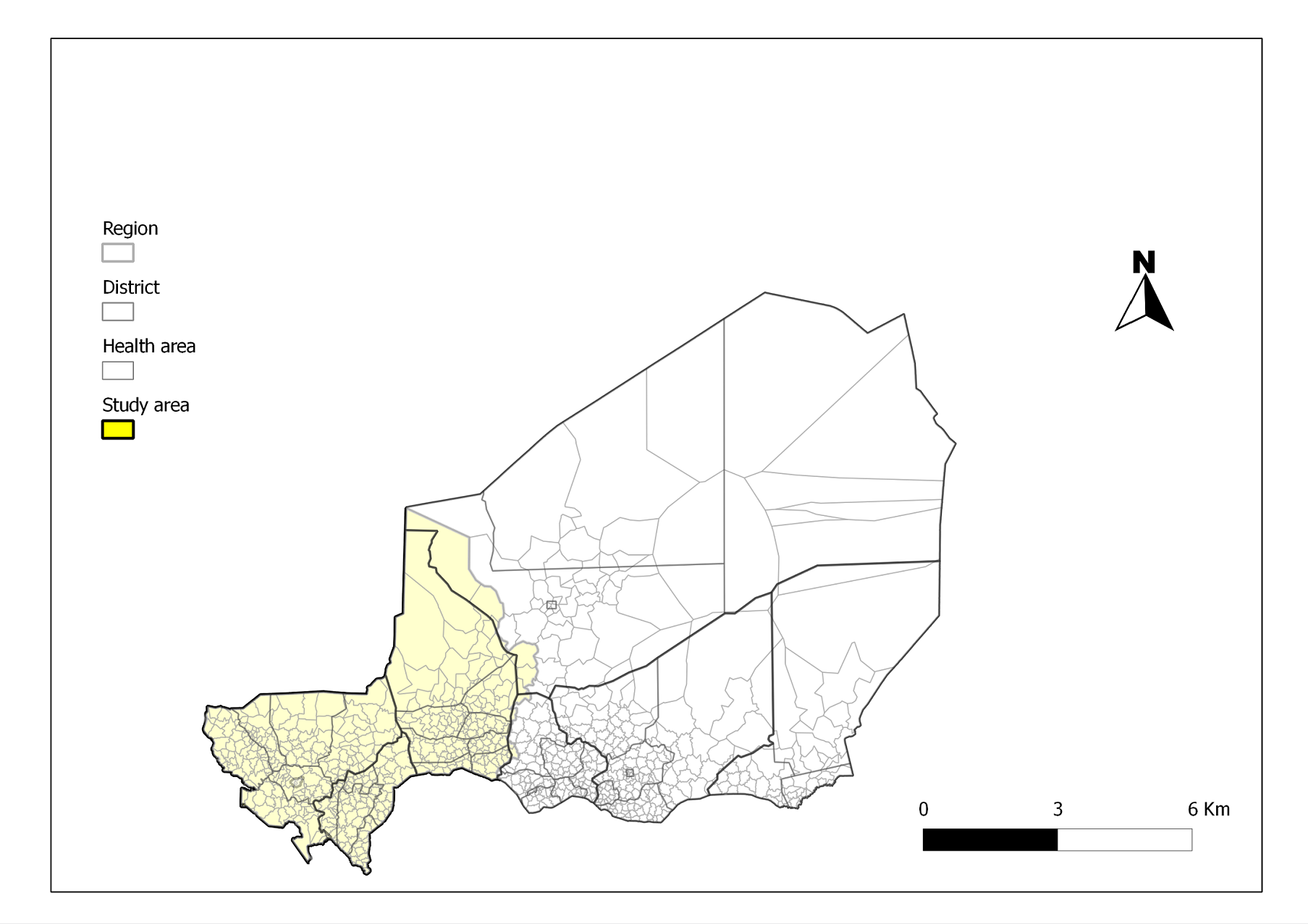

Supplement: S1 Fig — The yellow part corresponds to the study area and encompasses the three regions of Tahoua, Tillabery and Dosso. Health areas were defined based on the geographical locations of health centers and their surrounding villages, and therefore do not always strictly match the borders of the administrative regions. (TIFF) [file pone.0163110.s003.tiff]
